# Exploring the effect of training community pharmacy staff in mentalization-based communication on recognizing patients' drug related problems: an uncontrolled pre-post intervention study in Denmark and the Netherlands

**DOI:** 10.1016/j.rcsop.2026.100731

**Published:** 2026-03-10

**Authors:** Ellen van Loon, Stijn Crutzen, Ramune Jacobsen, Ulla Hedegaard, Marcia Vervloet, Laura Schackmann, Liset van Dijk, Susanne Kaae, Katja Taxis

**Affiliations:** a Faculty of Science and Engineering, Unit of Pharmacotherapy, -Epidemiology and – Economy, Groningen Research Institute of Pharmacy, University of Groningen, Antonius Deusinglaan 1, 9713, AV, Groningen, the Netherlands; b Pharmacy De Drie Stellingen, Brink 1-101, 8431 LD Oosterwolde, the Netherlands; c Department of Pharmacy, Faculty of Health and Medical Sciences, University of Copenhagen, Universitetsparken 2, 2100 København Ø, Denmark; d Clinical Pharmacology, Pharmacy and Environmental Medicine, Department of Public Health, University of Southern Denmark, 5230 Odense, Denmark; e Nivel, Netherlands Institute for Health Services Research, Otterstraat 118, 3513 CR Utrecht, the Netherlands

**Keywords:** Drug-related problems, Medication safety, Community pharmacy, Patient-centered communication, Mentalizing

## Abstract

**Objective:**

To explore the effect of a comprehensive mentalizing education programme on pharmacy staff's ability to recognize drug related problems (DRPs) during counter conversations in community pharmacies.

**Methods:**

A multicentre, uncontrolled pre-post-intervention study was conducted in Danish and Dutch pharmacies. Over four months, pharmacists and pharmacy technicians completed a parttime mentalizing education programme. Participants documented all DRPs they recognized during six hours of counter conversations before and after the intervention. Data were analysed using mixed multilevel logistic regression, including a post-hoc comparison between countries.

**Results:**

Forty-one participants from twenty pharmacies registered 2507 conversations. The overall increase of recognized DRP frequency from 17.5% to 22.1% was not significant (*p* = 0.086). Post-hoc analysis showed a significant increase in the Netherlands (10.6%, *p* = 0.04). Recognized DRP-categories ‘compliance’, ‘monitoring’, and ‘education or information’ increased, while ‘drug selection’, ‘over/under dose’, and ‘toxicity/adverse drug event’ decreased. The category ‘undertreated’ decreased in Denmark, but increased in the Netherlands. In Denmark, referrals to other healthcare providers dropped from 44.8% to 21.4%.

**Conclusion:**

The mentalizing education programme shows promise to support pharmacy staff in recognizing and addressing DRPs, possibly through improved patient-centered communication.

## Highlights

• Pharmacy staff can play an important role in recognizing patients' drug-related problems (DRPs).
• Mentalizing is a promising concept that could potentially contribute to improve patient-centered communication.
• Enhanced mentalizing capacities may result in better recognition of DRPs at the pharmacy counter.
• Enhanced mentalizing capacities could support pharmacy staff to solve more DRPs themselves.

## 1 Introduction

Medication is effective in preventing and treating many diseases and can provide relief of burdensome symptoms. But despite proven efficacy, the potential benefits of medication are often not fully realized in clinical practice.^1^ Over two-thirds of patients experience drug related problems (DRPs), resulting in suboptimal outcomes, patient harm, and increased healthcare costs. DRPs include the use of inappropriate medication, dosage, administration, non-adherence, adverse drug reactions, drug-drug interactions, and contra-indications.^2^ Community pharmacy staff can play an important role in recognizing and solving DRPs because of their role of dispensing medication, their extensive knowledge of medication safety, and pharmacy's high number of daily encounters and good accessibility. Furthermore, computerized clinical decision support systems support pharmacy staff in DRP detection.^3^

It is widely acknowledged that patient-centered communication is crucial for identifying DRPs.4., 5 This is especially applicable for DRPs related to patients' experiences, needs and concerns. These issues vary from person to person and can only be revealed in a fruitful dialogue. However, conversations in community pharmacies are typically unidirectional, and focused on providing information rather than discussing patients' experiences and perceptions.^6^, 7., 8. Aspects such as non-adherence because of worries about medication use or motivational problems, inappropriate medication use or experienced side effects often remain overlooked. There are many barriers preventing better patient-centered communication in community pharmacies, including lack of privacy, high workload and a lack of patient's awareness of pharmacy's ability to assist with drug-related issues.^5^ Another important barrier is a lack of patient-centred communication skills of pharmacy staff. Pharmacy staff show emotional resistance when patients voice their unique perspectives. They attempt, most likely unconsciously, to avoid situations in which patients show negative emotions, because such interactions affect pharmacy staff emotionally.^9^

Patient-centered communication skills can be improved by training mentalizing skills of pharmacy staff.^9^ ‘Mentalizing’ is a psychological theory, referring to one's ability to understand one's own mental state (emotions, feelings, thoughts, and needs), the ability to understand the mental states of another and being aware of the effect on each other. Although everyone can mentalize to some extent, this capacity can be further developed through training.^10^, ^11^, ^12^ Given the emotional resistance of pharmacy staff to sharing personal perspectives from patients, strengthening mentalizing abilities should increase comprehension of patients. Pharmacy staff should be able to pay more attention to cues and make patients feel more comfortable sharing their medication-related problems. Staff should then also become better in understanding the impact of encounters on themselves, by considering their own mental state more appropriately. Training mentalizing is different from other communication trainings since it is based on curiosity and interest in one's own and other people's mental states, thereby addressing staff's attitudes as well as skills.^13^

Research on training healthcare providers in mentalizing is limited, but promising. Its primary application to date has been in the field of mental health, particularly among mental health clinicians^14^ and in people with intellectual disabilities.^15^ Training enhanced mentalizing capacities positively influenced health care providers' attitudes towards their clients.^14^, ^16^, ^17^ In Israel, integrating mentalizing into nursing curricula enhanced students' ability to see patients' perspectives, improved interpersonal skills, and supported professional identity formation.^18^

To explore the usefulness of mentalizing among pharmacy staff, Fosgerau et al. developed a continuing education programme to improve mentalizing skills and patient-centeredness in pharmacy counseling, spanning over four months.^13^ Evaluations showed that staff became better at understanding relevant mental states and communicating effectively, enabling them to focus on patients' perspectives.^19^ Video-observations revealed that after the education programme, patients expressed concerns more openly, and staff more frequently elicited and acknowledged patients' needs and concerns.^20^

**Fig. 1:**
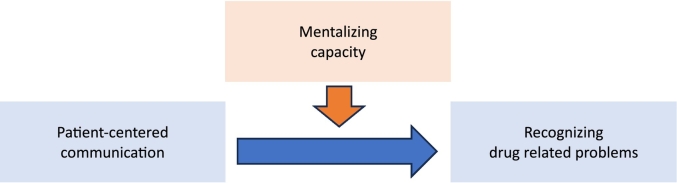
Improved mentalizing capacity may enhance patient-centered communication skills, so pharmacy staff can recognize and solve more drug related problems in conversations with patients at the pharmacy counter.

In this study, we want to further explore the relationship between improved mentalizing capacity and pharmacy staff's ability to recognize DRPs affecting patients' daily life as depicting in Fig. 1. The aim of this study is therefore to explore the effect of the mentalizing education programme on staff's ability to recognize DRPs during counter conversations in community pharmacies. Associated objectives are to explore the effect of the programme on the frequency and the type of recognized DRPs and the type of proposed solutions.

## 2 Methods

### 2.1 Setting and design

This was a multicentre uncontrolled pre-post-intervention study performed in Danish and Dutch community pharmacies.

Other quasi experimental study designs were considered, for example waitlist control, stepped-wedge design, or matched comparison pharmacies, but such designs were not feasible given the tight timeline and the available resources for the current study.

Both Denmark and the Netherlands provide a high level of pharmaceutical care compared to European standards.^6^, ^21^, ^22^ Pharmacy staff members in these countries primarily consist of pharmacists and technicians with different levels of training. Becoming a pharmacy technician takes three years in the Netherlands and three and a half years in Denmark, with additional training available in the Netherlands.23., 24. Patient counseling during dispensing is routine practice in both countries, and medication safety is supported by automated clinical decision support systems, such as dose and drug-drug-interaction checks. In both countries, the majority of regular counter conversations are performed by pharmacy technicians. Key differences exist between the countries in the extent of data exchange between general practices and pharmacies. In the Netherlands, clinical data such as laboratory values and medication dispensed in other pharmacies are accessible, unlike in Denmark. In Denmark, it is common for prescriptions to include the indication, printed on the patients' medication label alongside the dosing regimen. In the Netherlands, indications on prescriptions are required only for a limited number of medicines.

### 2.2 Intervention

The mentalizing education programme as described by Fosgerau et al.^13^ was developed in Denmark and piloted in Denmark and the Netherlands. Box 1 gives a more detailed description of the programme. The four-month programme ran from late September 2021 to early January 2022.

**Box 1:** Design of the mentalizing education programme.

Pharmacy staff was trained in ‘mentalizing communication’ and the ‘mentalizing mindset’, practicing patient-centeredness, through developing an open and curious stance towards patients as well as increased self-awareness. They were trained by a mentalizing expert (psychologist), supported by communication trainers who were pharmacists or researchers. The mentalizing education programme lasted four months and consisted of physical meetings and online meetings. The education consisted of theoretical background and models combined with exercises. Module 1: standards for counseling at the pharmacy; introduction to mentalizing and the mentalizing mindset; the importance of emotions in relation to behaviour; visit from patients – how do patients perceive their life with chronic illness; the affective-cognitive dimension. Module 2: the development of the capacity to mentalize; interpersonal differences in perspectives; how to express the mentalizing mindset; the self-other dimension; own communication tendencies and mentalizing capacity; individual feedback specific on mentalizing skills on video recordings of their own conversations with patients. Module 3: epistemic trust; how customers perceive the pharmacy; the implicit-explicit dimension. Module 4: the context dependency of the capacity to mentalize. Module 5: recap; practice through role-playing. Module 6: recap of key concepts; individual feedback on their own video recordings; reflection on own mentalizing potentials and barriers.

### 2.3 Recruitment of participants

Pharmacists and pharmacy technicians working in community pharmacies or outpatient pharmacies were eligible to participate in the study. We invited potential participants via e-mail and online promotion through the pharmacy networks of the researchers. The invitation was aimed at including two to three participants from each pharmacy, with preferably a combination of a pharmacist and one or two pharmacy technicians. Participation was free of charge, and participants committed to adhere to the course's scientific evaluation components.

### 2.4 Outcomes and definitions

The main outcome was the frequency of DRPs identified during counter conversations before and after the four-month mentalizing programme. A DRP was defined as ‘an event or circumstance involving drug treatment that actually or potentially interferes with the patient experiencing an optimal outcome of medical care’.^25^ Secondary outcomes were the frequency of types of DRPs and solutions identified.

DRPs were categorized using the validated DOCUMENT classification system,^26^ using the categories: drug selection, over or underdose prescribed, compliance, untreated indication, monitoring, education or information, non-clinical and toxicity or adverse reaction. Solutions that the pharmacy staff provided for the identified DRPs were based on DOCUMENT categories, grouped into: adjustment of the prescription, referral to physician, pharmacist or other, provided information, and other. The category ‘monitoring’ was added to provide room for follow-up. We adjusted the categorisation of solutions using groups and adding the category monitoring to make the registration as easy, comprehensive and fast as possible, thereby reducing the burden of data collection for participants.

### 2.5 Data collection

Participants completed a short questionnaire capturing demographic information (age, gender, education). To collect data on DRPs, participants registered information on conversations at the counter with patients when dispensing prescribed medicines for a duration of six hours pre- (September 2021) and six hours post-intervention (December 2021–January 2022). Six hours of data collection per participant were chosen as an optimal compromise between the required effort from participants and a sufficient volume of conversations for examining variations in DRP identification. Participants were allowed to register for several slots until they reached a total of six hours, as they usually did not conduct six consecutive hours of counter conversations. Conversations with relatives of a patient or conversations with patients under the age of eighteen were excluded, as well as conversations about over-the-counter medicines and non-medicines (like incontinence material or wound dressings), telephone conversations, and scheduled consultations.

For each conversation, a registration template was completed online or on paper and later digitized. The registration document is shown in Appendix A. Registration captured:

− participant's individual research code
− patient's age category and gender
− prescription type (first or repeated, i.e. patient had this medicine prescribed previously)
− whether a DRP was recognized

If a DRP was recognized during the counter conversation, additional information was registered:

− medication involved
− brief description of the problem (free text)
− DRP category
− solution type (possibility to choose multiple solutions)
− additional notes (optional)

DRPs which were recognized outside the counter conversation (e.g. if an interaction was discussed with the prescriber during processing electronically offered prescriptions) were not included as recognized DRP at the counter. If several DRPs were discussed in one conversation, then the most important DRP according to participants' opinion was registered.

Prior to the first data registration, participants were trained on DRP registration using the DOCUMENT system. Training included reading the manual, applying categories to 20 cases, and receiving feedback through meetings (Denmark) or via personal email (Netherlands). A help card with category examples supported staff during registration. Those documents are available on request from the authors.

In Denmark, staff obtained oral patient consent to collect DRP data, while in the Netherlands this was unnecessary as only anonymized data was recorded.

No formal sample size calculation was performed because of the exploratory nature of the study and the constraints in resources because of the scope of the study as highlighted above.

### 2.6 Analysis

Participants who registered fewer than five DRPs during six hours pre- or post-intervention were excluded. These participants were drop outs of the study, as they insufficiently engaged with the data collection process. Two researchers from each country (EL and SC in the Netherlands, UH and RJ in Denmark) quality-checked all registrations according to the DOCUMENT system.^26^ Errors in inclusion, DRP classifications, and registration were re-coded based on free-text data. Descriptive analyses were conducted for DRP frequency, types, and solutions.

The frequency of DRPs was determined by dividing the number of conversations with a DRP by the total number of conversations, expressed as percentage. The same was done for type of DRP and solutions.

To evaluate changes in DRP frequency and account for the nesting of measurements within participating staff, a mixed multilevel logistic regression model was used. DRP recognition (yes/no) was the dependent outcome, with participants treated as separate clusters. Intraclass correlation (ICC) determined differences in DRP recognition across participants. Random slopes and intercepts accounted for variations in baseline ability to recognize DRPs and training effects. Country (Netherlands/Denmark) was included as a fixed effect level 2 variable, and prescription type (first/repeat) was included as a fixed effect level 1 variable.

Chi-squared tests assessed whether patient age or gender influenced DRP detection, determining their inclusion in the model. A post-hoc analysis examined the interaction between country and education effect, as the increase of DRP detection rates differed between both countries.

In Denmark, DRPs without patient consent were included with missing details. A sensitivity analysis was performed in which these registrations were excluded.

Secondary outcomes were analysed descriptively.

Stata® version 18.5 was used to analyse the data.

### 2.7 Ethics and confidentiality

Participants received a unique research code for secure DRP registration (NL: Qualtrics; DK: SurveyXact). Data was securely stored at Groningen and Copenhagen universities and removed from online servers.

The Medical Ethics Review Board of the University Medical Center Groningen (METc UMCG) concluded that this study is not a clinical research with human subjects as meant in the Medical Research Involving Human Subjects Act (WMO) and that a review was therefore not necessary (reference number 202100510). In Denmark, data storage and processing were approved by the Data Protection Agency (handled by the Faculty of Health at the University of Copenhagen; reference number 514–0310/19–3000).

## 3 Results

A total of 50 pharmacy staff members who participated in the four-month mentalizing education were invited to register conversations at the pharmacy counter. Nine were excluded, as they registered less than five DRPs before, or after the education programme (DK 2 exclusions; NL 7 exclusions). As a result, DRP registrations were based on registrations from 41 participants (25 DK from 11 pharmacies and 16 NL from 9 pharmacies). Participant characteristics are presented in Table 1. All but one were female, most were pharmacy technicians, and the majority were aged 35–44 years. Age distribution was similar between Denmark and the Netherlands.

**Table 1:** Characteristics of the 41 participants in the mentalizing education programme who registered data on drug related problems.

	Mentalizing education participants *n* (%)		
	Denmark	Netherlands	Total
**Gender**			
Female	24 (96.0)	16 (100)	40 (97.6)
Male	1 (4.0)	0 (0.0)	1 (2.4)
**Age**			
<25	2 (8.0)	1 (6.3)	3 (7.3)
25–34	6 (24.0)	5 (31.3)	11 (26.8)
35–44	8 (32.0)	5 (31.3)	13 (31.7)
45–54	4 (16.0)	2 (12.5)	6 (14.6)
>54	5 (20.0)	1 (6.3)	6 (14.6)
unknown		2 (12.3)	2 (4.9)
**Occupation**			
Pharmacist	12 (48.0)	5 (31.3)	17 (41.5)
Pharmacy technician	13 (52.0)	11 (68.8)	24 (58.5)

Participants registered 2507 conversations: 1667 in Denmark (909 pre- and 758 post-intervention) and 840 in the Netherlands (452 pre- and 388 post-intervention). The intraclass correlation (ICC) was 0.10 [0.06–0.18]. Patient characteristics (Table 2) showed no significant differences in age or gender between countries or pre- and post-measurement (age: DK: *p* = 0.790; NL: *p* = 0.137; total: *p* = 0.282; gender: DK: *p* = 0.845; NL: *p* = 0.904; total: *p* = 0.819).

**Table 2:** Patient and prescription characteristics of registrations of the conversations.

	Before *n* (%)			After *n* (%)		
	DK	NL	Total	DK	NL	Total
**Age**						
> 80	80 (8.8)	40 (8.8)	120 (8.8)	89 (11.7)	35 (9.0)	124 (10.8)
71–80	226 (24.9)	95 (21.0)	321 (23.6)	151 (19.9)	83 (21.4)	234 (20.4)
61–70	191 (21.0)	107 (23.7)	298 (21.9)	156 (20.6)	78 (20.0)	234 (20.4)
51–60	165 (18.2)	76 (16.8)	241 (17.7)	139 (18.3)	76 (19.6)	215 (18.8)
41–50	90 (9.9)	55 (12.2)	145 (10.7)	79 (10.4)	41 (10.6)	120 (10.5)
31–40	76 (8.4)	36 (8.0)	112 (8.2)	63 (8.3)	36 (9.3)	99 (8.6)
21–30	55 (6.1)	29 (6.4)	84 (6.2)	55 (7.3)	21 (5.4)	76 (6.6)
18–20	26 (2.9)	14 (3.1)	40 (2.9)	26 (3.4)	18 (4.6)	44 (3.8)
Total	909 (100)	452 (100)	1361 (100)	758 (100)	388 (100)	1146 (100)
**Gender**						
Male	395 (43.5)	195 (43.1)	590 (43.4)	333 (43.9)	169 (43.6)	502 (43.8)
Female	514 (56.5)	257 (56.9)	771 (56.6)	425 (56.1)	219 (56.4)	644 (56.2)
**Type of prescription**						
First prescription	262 (28.8)	126 (27.9)	388 (28.5)	219 (28.9)	109 (28.1)	328 (28.6)
Repeated prescription	647 (71.2)	312 (69.0)	959 (70.5)	539 (71.1)	260 (67.0)	799 (69.7)
Combination first + repeated	–	14 (3.1)	14 (1.0)	–	19 (4.9)	19 (1.7)

### 3.1 Drug related problems

The quality-checking resulted in removal of 28 DRP registrations (DK 8; NL 20; overall 5.4%) because the registration did not meet the inclusion criteria. The DRP category was recoded in 123 cases (DK 76; NL 47; overall 25.1%).

A DRP was recognized in 17.5% of the counter conversations before and 22.1% of the counter conversations after the education programme (Table 3). The increase was not significant (*p* = 0.086, Table 4). Recognized DRPs were more than twice as likely with first prescriptions than repeat ones (OR 2.12, *p* ≤0.001), with no significant differences between the countries (*p* = 0.244, Table 4). Sensitivity analyses excluding 22 registrations lacking consent did not alter these results (Appendix B).

**Table 3:** Types of drug related problems (DRPs) identified during counter conversations before and after the intervention in Denmark (DK) and the Netherlands (NL).

	Before *n* (%)			After *n* (%)			Example
	DK	NL	Total	DK	NL	Total	
Total number of DRPs	159 (17.5)	79 (17.4)	238 (17.5)	144 (19.0)	109 (28.0)	253 (22.1)	
Drug selection	22 (13.8)	20 (25.3)	42 (17.6)	15 (10.4)	14 (12.8)	29 (11.5)	First prescription for itraconazole capsules. Because of drug interaction with azithromycin (Qt-prolongation) and recent heart surgery, the prescription was converted to miconazole oral gel.
Over- or underdose	18 (11.3)	12 (15.1)	30 (12.6)	12 (8.3)	10 (9.1)	22 (8.7)	Prednisolone phase-out schedule was unclear.
Compliance	36 (22.6)	11 (13.9)	47 (19.7)	35 (24.3)	17 (15.5)	52 (20.6)	Patient is very confused, request for multidose drug dispensing.
Undertreated	10 (6.3)	2 (2.5)	12 (5.0)	6 (4.2)	9 (8.2)	15 (5.9)	Triamcinolone cream prescribed for eczema, but no indifferent cream. Cetomacrogol ointment added.
Monitoring	6 (3.8)	0 (0.0)	6 (2.5)	10 (6.9)	6 (5.5)	16 (6.3)	When discussing the effect of levothyroxine, it comes to light that thyroid function hasn't been checked for years.
Education or information	31 (19.5)	19 (24.0)	50 (21.0)	34 (23.6)	32 (29.3)	66 (26.1)	It was unclear for the patient when the macrogol plus electrolytes should be taken for colonic lavage.
Toxicity or adverse drug event	17 (10.7)	15 (18.9)	32 (13.4)	9 (6.3)	18 (16.5)	27 (10.7)	Discussing the effect of linaclotide, patient tells the medicine does not yet what it is supposed to do, she mainly gets diarrhoea from it without it calming her intestines.
Not classifiable	7 (4.4)	0 (0.0)	7 (2.9)	14 (9.7)	3 (2.7)	17 (6.6)	
Missing data#	12 (7.5)	0 (0.0)	12 (5.0)	9 (6.3)	0 (0.0)	9 (3.6)	

# missing data: cases with a DRP in which the patient did not give consent for further registration.

**Table 4:** Multilevel logistic regression of the identified drug related problems.

Main analysis				
	Odds ratio	Standard error	P-value	95% CI
Before/after measurement	1.28	0.19	0.086	[0.97–1.71]
Prescription type (first prescription = 1)	2.12	0.25	<0.001	[1.69–2.67]
Netherlands/Denmark (NL = 1)	1.37	0.37	0.244	[0.81–2.31]
**Post-hoc analysis**				
Before/after measurement	1.04	0.18	0.800	[0.74–1.47]
Prescription type (first prescription = 1)	2.07	0.24	<0.001	[1.66–2.59]
Country (NL = 1)	1.03	0.28	0.611	[0.61–1.74]
Before/after Country	1.74	0.48	0.044	[1.02–2.97]

In Denmark, a DRP was identified in 17.5% and 19.0% of conversations pre- and post-intervention. In the Netherlands, a DRP was identified in 17.4% and 28.0% of conversations pre- and post-intervention (Table 3). Therefore, a post-hoc analysis with added interaction between the education programme and country was performed. This analysis showed that there was a statistically significant increase in the frequency of DRPs in the Netherlands (OR 1.74, *p* = 0.044, Table 4).

The most common identified DRP categories were ‘education or information’ and ‘compliance’, comprising 45% of all recognized DRPs (Table 3). The relative recognition of DRPs in the following categories increased in both countries: ‘compliance’, ‘monitoring’, and ‘education’, whereas it decreased in the following categories: ‘drug selection’, ‘over or under dose’, and ‘toxicity or adverse drug event’. The percentage of the category ‘undertreated’ relatively decreased in Denmark, but increased in the Netherlands (6.3% to 4.2% versus 2.5% to 8.2%, respectively).

### 3.2 Solutions of DRPs

Solutions that the pharmacy staff provided for DRPs showed most often ‘providing information’ (Table 5). Cases in which ‘providing information’ was chosen as the solution, without the addition of ‘referring to another healthcare provider’, was observed more often after the education programme in Denmark (from 71/147 (48.3%) to 93/135 (68.9%)), but less often in the Netherlands (from 47/79 (59.5%) to 60/109 (55.0%)). ‘Adjustment of prescriptions’ and ‘referral to other healthcare providers’ was observed less frequently after the education programme (16.8% to 12.7% for prescription adjustments, and 34.5% to 20.0% for referrals). This shift was observed more often in Denmark than in the Netherlands. In the Netherlands, the category ‘monitoring’ showed the largest shift.

**Table 5:** Solutions that the pharmacy staff provided for the identified drug related problems (DRP).

	Before *n* (%)			After *n* (%)		
	DK	NL	Total	DK	NL	Total
**Complete DRP registrations**	147	79	226	135	109	244
**Solution**#						
Adjustment of the prescription	18 (12.2)	20 (25.3)	38 (16.8)	7 (5.1)	24 (22.0)	31 (12.7)
Referral to physician, pharmacist or other	66 (44.8)	12 (15.1)	78 (34.5)	29 (21.4)	20 (18.3)	49 (20.0)
Provided information	115 (78.2)	48 (60.7)	163 (72.1)	110 (81.4)	67 (61.4)	177 (72.5)
Monitoring	8 (5.4)	2 (2.5)	10 (4.4)	9 (6.6)	10 (9.1)	19 (7.7)
Other	7 (4.7)	0 (0.0)	7 (3.1)	7 (5.1)	0 (0.0)	7 (2.9)

# Participants could register more than one solution per DRP.

## 4 Discussion and conclusion

In this exploratory study, pharmacy staff trained in mentalization-based communication identified a non-significant increase in DRP recognition during regular counter conversations after the mentalizing programme. An exploratory post-hoc analysis showed that this was significant for pharmacy staff in the Netherlands. Although the numbers of the separate categories are too small to draw firm conclusions, the descriptive analysis of types of recognized DRPs suggests small shifts. Danish staff relatively solved more DRPs themselves instead of referring to other healthcare providers after the programme. Although less pronounced, in the Netherlands there was a relative move towards monitoring. The absence of a control group means that these results need to be interpreted cautiously in the light of the many different factors potentially impacting the outcome which we will address in this discussion.

### 4.1 Discussion

In previous studies, it has already been shown that the four-month mentalizing education programme improved the mentalizing capacities of pharmacy staff.^13^, ^19^ Video analyses of conversations before and after the mentalizing programme showed that patients appeared to express their concerns more explicitly, and pharmacy staff more often elicited and recognized patients' needs and concerns.^20^ The current study suggests that improved mentalizing capacities can enhance pharmacotherapeutic outcomes, as pharmacy staff identified more DRPs during regular counter conversations.

Before the programme, a DRP was identified in about 17% of all pharmacy counter conversations in both nations, which was a remarkably high baseline level. Detection rates for DRPs in community pharmacies from other research in the Netherlands, Germany, Sweden, and Switzerland range from 1.8 to 22.6%,^27^, ^28^, ^29^, 30. with each study employing a distinct set of inclusion criteria and methodology. Unlike these studies, our baseline included only DRPs identified during counter conversations, excluding DRPs of over-the-counter medicines and those detected elsewhere in the process, such as during prescription processing. The high baseline might reflect participants' deliberate focus on DRPs during conversations, a potential effect of the self-reporting method. Nevertheless, in the Netherlands the rate of recognizing DRPs significantly increased towards 28.0%, while in Denmark there was no significant increase. This may indicate that the staff's ability to recognize DRPs during counter conversations in community pharmacy increased due to the mentalizing programme in the Netherlands. Although there were no major shifts in DRP type, categories related to patients (‘compliance’ and ‘education or information’) relatively increased, while more drug-related categories (‘drug selection’ and ‘over- or underdose’) showed a relative decrease. This trend was slightly more noticeable in the Netherlands, which aligns with improved mentalizing skills, as patient-related DRPs often require patient interactions to uncover.

There is no clear explanation for the different outcomes in the two countries, but several factors may contribute. First, a possible reason is the proportion of participating pharmacists and pharmacy technicians. The Netherlands had relatively more technicians. It is possible that the mentalizing course may have been more beneficial for technicians than pharmacists. Second, there was a higher dropout rate in the Netherlands than in Denmark (NL 7; DK 2). We have no clear explanation for this difference. Of note, the study was performed in 2021/22 which was during the COVID-19 pandemic. This posed many challenges for health professionals and may have affected community pharmacy staff in the Netherlands differently from Denmark. Possibly staff which was more dedicated to the education remained in the project. A third explanation might be the difference in access to clinical data between both countries. Because the trend towards more patient-related categories was somewhat stronger in the Netherlands, mentalizing may have enabled pharmacy staff to better incorporate clinical data into conversations, leading to recognizing more DRPs. Finally, differences in the educational programme could have influenced outcomes. In the Netherlands, participants received personal feedback on the exercise in recognizing DRPs via e-mail prior to the premeasurement, while in Denmark, exercises were discussed centrally. The individual comments might have enhanced understanding and recognition of DRPs, although the expected difference in the pre-measurement was not observed.

There were interesting differences in DRP solutions between the two countries. Danish participants resolved relatively more identified DRPs by ‘providing information’ rather than referring to other healthcare providers after the mentalizing programme. This could suggest a trend of increased patient-centeredness and may mean that pharmacy staff became more self-assured, showing more interest and acceptance of patients. Hereby according to theory, trust is created,^19^ and more relevant information from the patient to be uncovered and addressed. As shown in other evaluation data in Denmark, mentalizing education led to significant improvements in awareness of both their own and the patients' emotional states.^19^ This trend was not observed in the Dutch results, where referrals were already low at baseline. In the Netherlands, there was a less noticeable relative shift towards increased monitoring, which may also indicate increased patient-centeredness. Perhaps the difference of available clinical data can play a role, as Dutch pharmacy staff may have more opportunities to manage DRPs in other ways. But given the descriptive analysis of the data on shifts in categories and the lack of definite patient outcomes those results need to be interpreted cautiously.

In first prescriptions it was more than twice as likely to recognize DRPs compared to repeated prescriptions. This study examined regular counter conversations. During these conversations, there is usually limited interaction regarding repeated prescriptions and patients' needs and concerns about medication are most often not explored.7., 31 This is unfortunate, as mentalizing capacities can be especially beneficial for conversations about repeated prescriptions, focusing on the experiences using the medications chronically. In those situations, having an open and inquisitive mindset is essential.

### 4.2 Strengths and limitations

The study design comes with strengths and limitations. This is a unique study in investigating the effect of a novel education in two countries on a relevant outcome related to patient-centered communication. The study involved a multicentre sample, enhancing the reliability and generalisability of the research findings. Furthermore, it was valuable to assess the mentalizing education programme's impact across all types of prescription dispense conversations, reflecting its potential in routine pharmacy operations. Exploratory work, like our study, is an important step in the development of health care interventions, particularly valuable in understanding the context and potential effects of novel interventions.^32^ Some limitations need to be addressed. First, a major limitation is the lack of blinding and control which makes it impossible to disentangle the effects of the programme from other effects during the study period. Inclusion bias cannot be ruled out, and participants may have unconsciously increased their efforts to detect DRPs (the so-called Hawthorne effect). Additionally, by measuring DRPs before the education programme, DRPs could have become a topic that was more often discussed in the participating pharmacies. The high number of DRPs could be a result of participating in the programme, a learning effect (or response-shift bias) which is particularly relevant for educational interventions like ours. Second, our outcome measure relied on self-registration during routine work. Lack of time and lack of training are important barriers for detecting and classifying DRPs.^33^ Although staff were trained in using the categorisation system in this study, this may have been insufficient to apply all categories consistently. In addition, staff may have completed recordings under time pressure, as the DRP-registrations had to be completed during their regular work. Fatigue may therefore have affected the quality of registration, although this should be the same in both measurement periods. We did take quality assurance measures to minimise possible categorisation bias resulting in recoding about 25% of DRPs. But we did not take additional measures such as calculating kappa values to assess the reliability of this process. More work is needed to develop easy to use, standardised approaches to measure DRPs in routine practice. Third, only one DRP per conversation was registered, even when multiple DRPs were discussed in a conversation. We have therefore likely underestimated the total number of DRPs and were not able to detect a possible shift in the number of DRPs per conversation. This may also cause bias in the distribution of types of DRP. Future work could investigate whether mentalizing may lead to identifying more (and other) DRPs per conversation. Fourth, we were not able to include other potentially relevant covariates in our analysis such as staff role (pharmacists and pharmacy technicians), type of pharmacy, workload, or baseline DRP rate, as this was beyond the scope of the study. Such data would have been valuable for positioning of future mentalizing training programmes and is of interest for future research, especially since delegating counter conversations to pharmacy technicians is not common practice in all countries. A final important limitation to mention is that we focused on the frequency and type of DRPs. While DRPs are an important intermediate outcome, it is unknown whether recognizing more DRPs is associated with improved patient-level outcomes such as medication safety, adherence or patient well-being. The impact of DRPs from a patient perspective and relevance regarding clinical outcomes remain unknown.

### 4.3 Implications and recommendations

Patients face challenges in receiving their medication, understanding how or why to take their medication, and obtaining satisfactory effects.^34^ We saw that in a significant proportion of the regular counter conversations a DRP was identified, highlighting the counter conversation as an important moment to address DRPs. Pharmacy staff needs to be better equipped to transition from technical instructions towards patient-centered conversations and guidance of patients encountering DRPs. The results of this study together with the related video analysis study^19^ should be regarded as an encouragement for further research into the effects of enhanced mentalizing capacities on the quality of care in community pharmacy over the longer term. The study period was relatively short. Patients are accustomed to relatively superficial and instructional interactions in the pharmacy setting. It takes time to engage them in reconceptualising the pharmacy as an appropriate and accessible point of contact for discussing experiences with medication use. The same applies to pharmacy staff. Our study is a good basis to inform future studies in line with guidance to develop complex interventions,^33^ ideally using more robust designs. Such studies need to combine qualitative and quantitative measures over a longer period and include relevant clinical and patient reported outcome measures as highlighted above. Given the exploratory findings on possible country-specific effects, cross national studies may provide additional insights. Additionally, evaluating increased mentalizing capacities for pharmacists versus technicians would be valuable, as technicians interact with patients more frequently, while pharmacists handle more complex cases. Furthermore, assessing the effect of mentalizing skills on other types of conversations in the pharmacy, such as longer separate self-care consultations and medication reviews, would provide further insights.

### 4.4 Conclusion

The new four-month continuing education programme in mentalizing among pharmacy staff in Denmark and the Netherlands did not significantly change the overall rate of recognizing DRPs, but post hoc analysis suggested that staff in the Netherlands may have recognized more DRPs. How staff solved DRPs seemed to shift, as relatively more DRPs were resolved without referring to other healthcare providers in Denmark. Therefore, this programme shows some promise to support pharmacy staff in patient-centered communication.

## Data access

Authors have collected all data directly from participants and have complete access which is ongoing.

## CRediT authorship contribution statement

**Ellen van Loon:** Writing – original draft, Visualization, Validation, Project administration, Methodology, Investigation, Formal analysis, Data curation, Conceptualization. **Stijn Crutzen:** Writing – original draft, Validation, Methodology, Investigation, Formal analysis, Data curation, Conceptualization. **Ramune Jacobsen:** Writing – review & editing, Validation, Project administration, Methodology, Investigation, Formal analysis, Data curation, Conceptualization. **Ulla Hedegaard:** Writing – review & editing, Validation, Methodology, Conceptualization. **Marcia Vervloet:** Writing – review & editing, Methodology, Conceptualization. **Laura Schackmann:** Writing – review & editing, Methodology, Conceptualization. **Liset van Dijk:** Writing – review & editing, Methodology, Conceptualization. **Susanne Kaae:** Writing – review & editing, Visualization, Supervision, Methodology, Investigation, Funding acquisition, Data curation, Conceptualization. **Katja Taxis:** Writing – review & editing, Visualization, Supervision, Methodology, Funding acquisition, Formal analysis, Data curation, Conceptualization.

## Funding

This work was supported by 10.13039/100014419EIT Health [ID 210638, ‘Patient Centered Communication in Community Pharmacy’].

## Declaration of competing interest

The authors declare that there are no conflicts of interest.

## Figures and Tables

**Table:** 

**FILL IN FOR ALL CONTACTS**				**ONLY FILL IN IF YOU RECOGNIZED A DRP**				
Age patient	Gender patient m/f	Type of prescription 1: first prescription(s) 2: repeated prescription(s) 3: combination first and repeated prescription(s)	did you RECOGNIZE a drp? yes/no	medicine(s) involved	brief description of the situation and the (potential) drp	drp category 1/2/3/4/5/6/7/8 (see assist card for classification)	solution 1/2/3/4/5 (multiple options possible) see assist card for classification	additional information you want to give (optional)

**Table:** 

Primary analysis: DK-registrations without consent included with missing details		Odds ratio	Standard error	p-value	95% CI
	Before/after measurement	1.28	0.19	0.086	[0.97–1.71]
	Prescription type (first prescription = 1)	2.12	0.25	<0.001	[1.69–2.67]
	Netherlands/Denmark (NL = 1)	1.37	0.37	0.244	[0.81–2.31]
Variation: excluding DK-registrations without consent		***Odds ratio***	***Standard error***	***p-value***	***95% CI***
	Before/after measurement	1.27	0.18	0.080	[0.97–1.68]
	Prescription type (first prescription = 1)	2.08	0.24	<0.001	[1.66–2.60]
	Netherlands/Denmark (NL = 1)	1.24	0.31	0.240	[0.76–2.02]

## Data Availability

The data underlying this article cannot be shared publicly due to the privacy of the individuals that participated in the study. All data will be shared on reasonable request to the corresponding author.

## Appendix A Registration template

Registration Drug Related Problems.

Date:
